# Hyperactive when young, hypoactive and overweight when aged: Connecting the dots in the story about locomotor activity, body mass, and aging in *Trpv1* knockout mice

**DOI:** 10.18632/aging.100306

**Published:** 2011-04-07

**Authors:** Samuel P. Wanner, Andras Garami, Andrej A. Romanovsky

**Affiliations:** ^1^ Systemic Inflammation Laboratory (FeverLab), Trauma Research, St. Joseph's Hospital and Medical Center, Phoenix, AZ 85013, USA; ^2^ Department of Physiology and Biophysics, Federal University of Minas Gerais, Belo Horizonte, Minas Gerais 31270-901, Brazil; ^3^ Department of Pathophysiology and Gerontology, Medical School, University of Pécs, H-7624 Pécs, Hungary

**Keywords:** Locomotion, obesity, body temperature, circadian rhythms, aging, vanilloid receptor

## Abstract

We have recently found that, at a young age, transient receptor potential vanilloid-1 (*Trpv1*) knockout (^−^/^−^) mice have a higher locomotor activity than their wild-type littermates (^+^/^+^). We have also found that, with age, *Trpv1*^−/−^ mice become substantially heavier than *Trpv1*^+/+^ controls, thus forming a paradoxical association between locomotor hyperactivity and overweight. The present study solves this contradiction. By using two experimental paradigms, we show that aged *Trpv1*^−/−^ mice have not an increased, but a decreased, locomotor activity, as compared to age-matched *Trpv1*^+/+^ controls. We also confirm that aged *Trpv1*^−/−^ mice are overweight. We conclude that TRPV1 channels are involved in the regulation of both general locomotor activity and body mass in an age-dependent manner.

## Two unexpected findings in Trpv1 knockout mice

Recent studies have shown that transient receptor potential (TRP) channels, including the vanilloid-1 channel (TRPV1), are involved in the regulation of multiple physiological functions in health and disease [1-3]. While studying the thermoregulatory phenotype of mice genetically deficient in TRPV1 channels (*Trpv1*^−/−^), we made two unexpected observations [4]. First, we found that young (age of ~17 wk; body mass of ~30 g) male *Trpv1*^−/−^ mice expressed a higher locomotor activity in a thermogradient apparatus, especially during the light (inactive) phase, as compared to their wild-type littermates (*Trpv1^+/+^*). In this apparatus, each mouse could move freely inside a 200-cm-long chamber with a linear ambient-temperature gradient from 20 to 30°C. The magnitude of the hyperactivity of *Trpv1*^−/−^ mice in this thermogradient apparatus was remarkable: at one time point during the inactive phase (3:00 P.M.), *Trpv1*^−/−^ mice were moving with an average velocity of 85 cm/min (> 1.2 km/day), which was ~3 times higher than the average speed of wild-type mice at the same time of day.

Our second unforeseen observation was that, with age, *Trpv1*^−/−^ mice of both sexes became heavier than their *Trpv1^+/+^* counterparts. For example, at the age of ~36 wk, the body mass of male *Trpv1*^−/−^ mice was 14% higher than that of the age-matched male controls: 49 ± 1 *vs* 43 ± 1 g (*P* < 1 × 10^−4^). The two heaviest mice in our colony were *Trpv1*^−/−^ males: one had a body mass of 59 g at the age of 35 wk, and the other weighed 58 g at 61 wk. Even though we did not measure the amount of fat in aged *Trpv1*^−/−^ mice, their phenotype was consistent with obesity. The animals had larger bellies, which suggested visceral fat accumulation, a generally accepted hallmark of aging [5]. We also noticed both a large amount of visceral fat and a thick layer of subcutaneous fat in aged *Trpv1*^−/−^ mice while performing surgeries for a different study (S. P. Wanner and A. A. Romanovsky, unpublished observations), as well as for the experiments reported in the present paper.

## Hyperactive when young

The finding of increased locomotor activity of young *Trpv1*^−/−^ mice contradicts several studies showing that the deletion of the Trpv1 gene does not affect the locomotor activity [6-8]. However, all these studies used much shorter observation periods, sometimes as short as 15 min. Furthermore, even though these studies found no statistically significant differences in the locomotor activity of *Trpv1*^−/−^ mice, some of them showed strong tendencies that would agree with our results [4]. For example, Davis et al. [6] found that *Trpv1*^−/−^ mice tended to have higher scores in several activity tests. An increased activity of *Trpv1*^−/−^ mice was also observed by Marsch et al. [8] in tests for anxiety-related behaviors. The increased spontaneous locomotor activity observed in our study [4] was unlikely to be due to changes in either anxiety or exploratory behavior, because the mice were extensively adapted to the experimental setups and were observed over long periods of time. Whether it was related to changes in the sleep pattern is unknown, but at least one highly potent and selective TRPV1 antagonist, BCTC, did not affect sleep in the study by Takeda et al. [9]. We hypothesized that TRPV1 channels were involved in a mechanism inhibiting the locomotor activity. We then confirmed this hypothesis in pharmacological experiments using central and peripheral administration of TRPV1 agonists and an antagonist in both Trpv1^+/+^ and *Trpv1*^−/−^ animals [4]. Hence, our experiments with genetic (*Trpv1*^−/−^ mice) and pharmacological tools suggest that TRPV1-mediated signals from the periphery tonically suppress general locomotor activity. Whether such suppression occurs due to a direct action on the locomotor system (i.e., on skeletal muscles or neural circuits involved in the locomotor control) or indirectly (*e.g*., by affecting motivation) remains to be elucidated.

## Overweight when aged

Our second observation — the predisposition of *Trpv1*^−/−^ mice to age-associated overweight — was also unexpected. It contradicted the studies by Davis et al. [6], Rong et al. [10], and Zhang et al. [11], who all found no changes in the body mass of *Trpv1*^−/−^ mice compared to controls, when maintained on either a regular or high-fat diet. Our observations also contradicted the study by Motter and Ahern [12], in which *Trpv1*^−/−^ mice were found less prone to become obese on a high-fat diet compared to wild-type controls. However, all abovementioned studies used young mice with the body mass in a 10-30 g range (on a regular diet). In this body mass range, the intergenotype difference in our study was also small, but with age (we observed the mice up to the age of 61 wk), the difference increased [4]. Furthermore, even though a higher body mass in aged *Trpv1*^−/−^ mice was not reported previously, the obesity-protective role of TRPV1 channels was proposed [11, 13] and supported by two lines of evidence. First, Zhang et al. [11] detected TRPV1 channels in preadipocytes, as well in murine and human visceral adipose tissue. These authors demonstrated TRPV1 downregulation during regular adipogenesis and found a reduced TRPV1 expression in visceral adipose tissue from obese mice and humans. Second, a long-term administration of capsaicin or capsinoids (nonpungent, capsaicin-related TRPV1 agonists) was shown repeatedly to suppress visceral fat accumulation, produce thermogenesis, and prevent an increase in the body mass in laboratory animals and humans [11, 14-17], possibly by stimulating catecholamine secretion [18], decreasing appetite and food intake [19], and increasing fat oxidation [14]. Interestingly, the loss of abdominal fat in obese humans that occurred in response to capsinoid treatment in the study by Snitker et al. [14] was associated with the Val585Ile *TRPV1* polymorphism: the subjects with the Val/Val and Val/Ile variants lost about twice as much abdominal fat as the study average, whereas Ile/Ile subjects lost none.

Cumulatively, the studies reviewed above, support the hypothesis that TRPV1 channels protect from aging-associated obesity.

## Hyperactive and overweight? Solving the paradox

**Figure 1.**
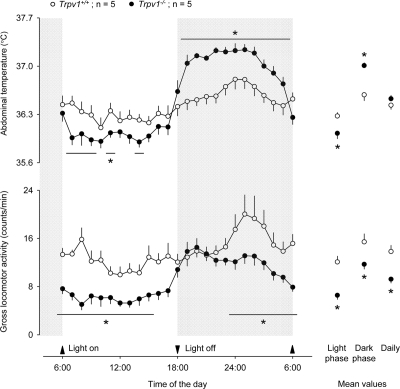
Compared to genetically unaltered controls, aged *Trpv1*^−/−^ mice have a suppressed locomotor activity throughout the day, a lower abdominal temperature during the inactive (light) phase, and a higher abdominal temperature during the active (dark) phase. The locomotor activity and abdominal temperature were measured by telemetry. The mice were kept in their home cages placed inside a climatic chamber; the ambient temperature was maintained at 28°C. The data are shown as means ± SE. Significant changes (*P* < 5 × 10^−2^, compared to *Trpv1*^+/+^ mice) are marked with asterisks (or horizontal lines with asterisks).

One paradox, however, remains unexplained. The association of locomotor hyperactivity (recorded at a younger age) with overweight (recorded at an older age) observed in our study [4] seems to contradict common sense. Obesity is known to be coupled with hyperactivity in certain pathological conditions, *e.g*., attention deficit hyperactivity disorder [20], but such coupling is rather an exception. To address this contradiction, we implanted miniature telemetry transmitters (G2 E-Mitter series; Mini Mitter) intraperitoneally in five aged male *Trpv1*^−/−^ mice (age: 44 ± 1 wk; body mass: 50 ± 2 g) and five aged male *Trpv1^+/+^* littermates (age: 43 ± 1 wk; body mass: 44 ± 1 g; all from the Amgen colony at Charles River Laboratories). All surgeries were performed as in our recent study [4], and all protocols were approved by the St. Joseph's Hospital and Medical Center Institutional Animal Care and Use Committee. On the third day after surgery, we measured the animals' body mass and then placed them into a climatic chamber (model 3940; Forma Scientific) with ambient temperature set to 28.0°C. In this setup, we studied their deep (abdominal) body temperature and locomotor activity for 48 h. The data obtained for each animal across the two days of observation were averaged and presented the means in Figure 1. The figure shows that the diurnal changes in body temperature of aged *Trpv1*^−/−^ mice are increased compared to their *Trpv1^+/+^* littermates, whereas their mean daily body temperature is unchanged. This is the same phenotype that was found in young *Trpv1*^−/−^ mice by Szelényi et al. [21] and, more recently, by Garami et al. [4]. However, even though the effects of the absence of TRPV1 channels on the body temperature of young and aged mice were the same, the effects on locomotor activity were the opposite. The gross locomotor activity of aged *Trpv1*^−/−^ mice was suppressed ' not exaggerated ' throughout the day, as compared to *Trpv1^+/+^*controls (9 ± 1 *vs* 14 ± 1 counts/min, *P* = 1 × 10^−3^).

**Figure 2.**
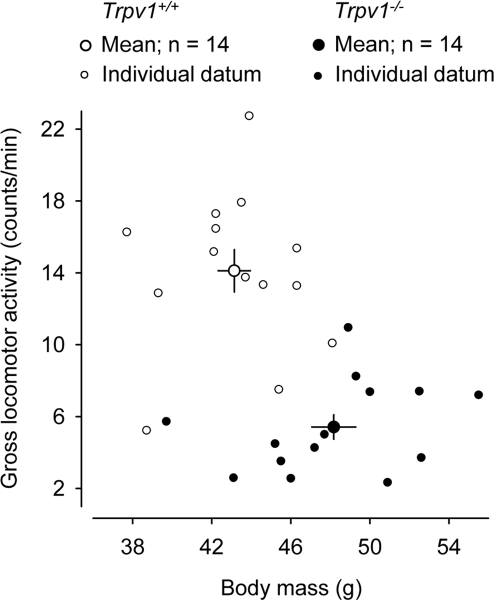
Aged *Trpv1*^−/−^ mice have a higher body mass (*P* = 1 × 10^−3^) and a lower general locomotor activity (*P* < 1 × 10^−7^) than their genetically unaltered littermates. Group means (± SE) and individual data are shown. The locomotor activity was measured by telemetry during a 3-h period at the beginning of the inactive phase. The ambient temperature was 28°C.

In addition to performing the measurements described above, we pooled together data from aged mice of both sexes, 14 *Trpv1*^−/−^ and 14 *Trpv1^+/+^*, used in a different study (S. P. Wanner and A. A. Romanovsky, unpublished observations). These data are plotted in Figure 2. The age in the two groups was identical (42 ± 1 wk), but the *Trpv1*^−/−^ were > 12% heavier than *Trpv1^+/+^* (48 ± 1 g *vs*. 43 ± 1 g; *P* = 1 × 10^−3^). The locomotor activity of these animals was measured for 3 h during the inactive phase (6:00-9:00 AM). This time period was chosen, because it corresponded to the maximum difference in the locomotor activity between aged *Trpv1*^−/−^ and *Trpv1^+/+^* mice (Figure 1). The methods of animal preparation and measurements were identical to those used to produce the results presented in Figure 1, and the protocols were approved by the same committee. The 3-h measurements in the additional animals (Figure 2) have confirmed that the gross locomotor activity of aged *Trpv1*^−/−^ mice is suppressed as compared to *Trpv1^+/+^* controls (5 ± 1 and 14 ± 1 counts/min, respectively; *P* < 1 × 10^−7^).

## Paradox solved

Hence, the paradox of non-matching locomotor activity and body mass has now been solved. When *Trpv1*^−/−^ mice are young, they show increased levels of activity, at least in some experimental paradigms [4]; at this age, they show no obesity [4, 6, 10-12]. When *Trpv1*^−/−^ mice age, they become hypoactive (present observations) and overweight (compared to wild-type littermates), as has been shown in our previous study [4] and the present study. Why *Trpv1*^−/−^ animals are hyperactive at a young age, but then start moving less (and gain body mass) is unclear. A similar relationship, however, was reported for a group of healthy humans [22]. In that group, those individuals who were more physically active at a young age showed a higher mass gain 11 years later. What is becoming clear is that TRPV1 channels are intimately involved in the regulation of both general locomotor activity and body mass, and that TRPV1-mediated regulation of both is age-dependent.
